# Fabrication and Characterization of Composite Biofilm of Konjac Glucomannan/Sodium Lignosulfonate/ε-Polylysine with Reinforced Mechanical Strength and Antibacterial Ability

**DOI:** 10.3390/polym13193367

**Published:** 2021-09-30

**Authors:** Xiaowei Xu, Jie Pang

**Affiliations:** School of Food Science, Fujian Agriculture and Forestry University, Fuzhou 350002, China; xxiaowei1995@163.com

**Keywords:** konjac glucomannan (KGM), KGM/SL/ε-P, composite film, food packaging

## Abstract

In order to enforce the mechanical strength and antibacterial ability of biofilm and explore the underlying mechanism, sodium lignosulfonate (SL) and ε-polylysine (ε-PL) were introduced to fabricate the composite film of konjac glucomannan (KGM)/SL/ε-PL in the present study. According to our previous method, 1% (*w*/*v*) of KGM was the optimal concentration for the film preparation method, on the basis of which the amount of SL and ε-PL were screened by mechanical properties enforcement of film. The structure, mechanical performance and thermal stability of the film were characterized by SEM, FTIR, TGA and tensile strength tests. The optimized composite film was comprised of KGM 1% (*w*/*v*), SL 0.2% (*w*/*v*), and ε-PL 0.375% (*w*/*v*). The tensile strength (105.97 ± 4.58 MPa, *p* < 0.05) and elongation at break (95.71 ± 5.02%, *p* < 0.05) of the KGM/SL/ε-PL composite film was greatly improved compared with that of KGM. Meanwhile, the thermal stability and antibacterial property of film were also enhanced by the presence of SL and ε-PL. In co-culturation mode, the KGM/SL/ε-PL composite film showed good inhibitory effect on *Escherichia coli* (22.50 ± 0.31 mm, *p* < 0.05) and *Staphylococcus aureus* (19.69 ± 0.36 mm, *p* < 0.05) by determining the inhibition zone diameter. It was revealed that KGM/SL/ε-PL composite film shows enhanced mechanical strength and reliable antibacterial activities and it could be a potential candidate in the field of food packaging.

## 1. Introduction

Konjac glucomannan (KGM) is a natural polymer found in konjac tubers showing unique physical and chemical properties because of its large molecular weight and complex chemical structure [1]. Compared with some other natural polymer materials, KGM has a larger molecular weight and a higher content of hydroxyl and acetyl groups. Therefore, KGM has stronger viscosity and hydrophilicity, and its hydrosol can form a dense and smooth film after drying [2]. KGM is widely used in some fields including the food packaging or medicine industry due to its good biocompatibility, biodegradability and good film-forming ability [3,4]. However, pure KGM films show several disadvantages such as poor mechanical properties and insufficient stability. In order to solve this problem, researchers have adopted a variety of methods to improve the mechanical strength, stability and other properties [5,6]. For example, it can be combined with other polysaccharides and proteins, metal nanoparticles, and reinforcing fibers (such as glass fibers, cellulose nanocrystals, and cellulose nanofibers) to improve performance [7,8,9,10]. However, with the large-scale use of metal nanoparticles and chemical synthetic fibers, this has also caused people to worry about the environmental pollution of metal nanoparticles. Some natural biological polymers such as lignin and its derivate could be environmentally friendly used for their easy aggregation to avoid pollution problems.

In this work, SL and ε-PL were introduced to improve mechanical strength, antibacterial ability and thermal properties of the KGM film as showed by performing tensile tests, TGA and antibacterial assessment. The structure of the developed films was evaluated by FTIR and SEM analysis. Lignin, as a rich biomass resource of plants, mainly exists in the cell walls of plants which improve the hardness and strength of plant tissues by virtue of the three-dimensional network structure [11,12]. Lignin is the third most amount of carbohydrate only next to cellulose and chitin. However, lignin, a very complex structure existing in a large number of plants, is generally discarded as waste. Over the recent decades, the high-value utilization of lignin has received extensive attention from researchers [13,14,15,16], who mainly focused on the application of lignin and its derivatives. The rational development and utilization of lignin has important research significance and conforms to the concept of sustainable development [17,18]. Lignosulfonate is a derivative of lignin extracted from liquid waste during pulping and papermaking processes [19,20,21]. Lignosulfonate is utilized as dispersants, water reducers and other low value-added products. In recent years, lignosulfonate was used as an anti-ultraviolet additive, a strengthening agent for films and gel materials in high-quality applications [22,23,24,25]. These unique properties of lignosulfonate are utilized to overcome the shortcomings of poor mechanical strength of konjac glucomannan, which is beneficial to the development of low-cost, high-performance composite materials.

ε-PL is a high molecular peptide with antibacterial effect on common bacteria, such as *Staphylococcus aureus* and *Escherichia coli*. ε-PL destroyed the cell membrane structure of microorganisms by interruption of cell material, energy and information transmission, and ultimately leading to cell death [26,27]. Therefore, ε-PL is a good food preservative and antibacterial agent [28,29]. 

**Figure 1:**
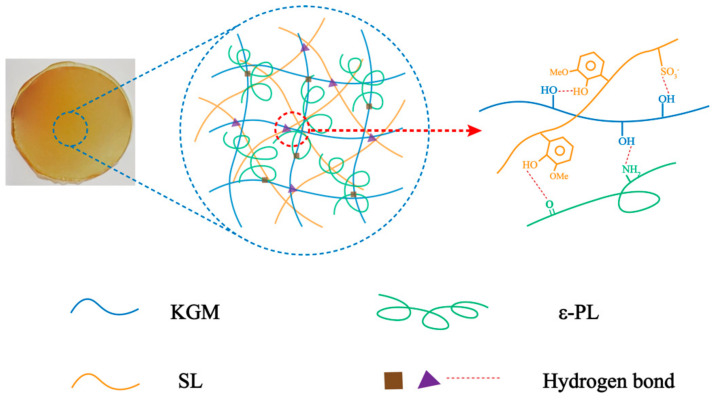
Mechanism diagram.

In this work, SL and ε-PL were introduced to improve the mechanical strength, antibacterial ability and thermal properties of the KGM film. The structure and performance of the film were characterized by SEM, FTIR, TGA and mechanical performance tests. The underlying mechanism of gel formation among KGM, SL and ε-PL was explored, Figure 1 shows the hydrogen bonding between polymer chains in detail. The antibacterial activities against the growth of *Escherichia coli* and *Staphylococcus aureus* were evaluated by the Oxford Cup test. The excellent performance of KGM/SL/ε-PL composite film was proved, and it could be a potential candidate for food packaging.

## 2. Materials and Methods

### 2.1. Materials 

Konjac glucomannan (KGM, 95%) was purchased from Yunnan Zhaotong San’ai Organic Konjac Development Co., Ltd. (Zhaotong, China). Both sodium lignosulfonate (SL) and ε-polylysine (ε-PL) were obtained from Shanghai Aladdin Biochemical Technology Co., Ltd (Shanghai, China). Glycerin and Na_2_CO_3_ (AR) were purchased from Sinopharm Chemical Reagent Co., Ltd (Shanghai, China). Deionized water was used in all experiments. *Escherichia coli* (ATCC 11775) and *Staphylococcus aureus* (ATCC 27735) were obtained from the School of Food Science, Fujian Agriculture and Forestry University (Fuzhou, China).

### 2.2. Preparation of KGM Films

According to the procedures of preparing KGM solution in our research group, KGM powder (1.00 g) was dissolved into 100 mL deionized water while mechanically stirred for 1 h to and thoroughly swelled to obtain a KGM control solution [30,31]. The solution was centrifuged at 3000 rpm for 6 min to be degassed. The centrifuged solution (30 mL) was poured into a petri dish (Φ 90 mm). After being wrapped in plastic with holes to allow water to evaporate, the solution was dried in a 50 °C oven to obtain intact KGM film.

### 2.3. Preparation of KGM/SL Films

After the addition of 0.10 g SL powder into 100 mL deionized water, the solution was mechanically stirred for 10 min to obtain 0.1% SL solution. Then 1.00 g KGM powder was added into above 0.1% SL solution and was thoroughly swelled with another 1 h agitation to obtain 0.1% mixed KGM/SL solution. When the serial amounts of SL powder 0.20, 0.30, 0.40, and 0.50 g were added to obtain 0.2, 0.3, 0.4, and 0.5% mixed KGM/SL solutions respectively. The solutions were centrifuged at 3000 rpm for 6 min to degas [32]. Each centrifuged solution (30 mL) was poured into a petri dish (Φ 90 mm) which was wrapped by plastic with holes to allow water to evaporate. The mixed solution was dried in oven (50 °C) to obtain intact (0.1, 0.2, 0.3, 0.4, and 0.5%) KGM/SL composite films. The mechanical strength of all the prepared films were evaluated as the method in 2.5. The composite film of 0.2% KGM/SL exhibited the strongest mechanical strength, so 0.2% SL was chosen as the optimal amount to be used for the following experiment.

### 2.4. Preparation of KGM/SL/ε-PL Films

After the addition of 0.20 g SL and 0.125 g ε-PL powder into 100 mL deionized water respectively, the solution was mechanically stirred for 10 min to obtain 0.2% SL and 0.125% ε-PL solution. Then 1.00 g KGM powder was added into the above solution and was thoroughly swelled with another 1 h agitation to obtain mixed KGM/SL/ε-PL solution with 0.125% ε-PL. When varied the amount of ε-PL powder was as 0.25, 0.375 and 0.50 g to obtain 0.25, 0.375 and 0.5% mixed KGM/SL/ε-PL solutions, respectively. The solutions were centrifuged at 3000 rpm for 6 min to degas. Each centrifuged solution (30 mL) was poured into a petri dish (Φ 90 mm) which was wrapped by plastic with holes to allow water to evaporate. The mixed solution was dried in an oven (50 °C) to obtain intact (0.125, 0.25, 0.375 and 0.5%) KGM/SL/ε-PL composite films. The mechanical strength of all prepared films was evaluated as the method in 2.5. The composite film of KGM/SL/ε-PL with 0.2% SL and 0.375% ε-PL exhibited the strongest mechanical strength and elongation at break, so 0.2% SL and 0.375% ε-PL were chosen as the optimal amount to be used for the following experiment.

### 2.5. Mechanical Strength Evaluation of Films

Those pieces free of defects (mainly no bubbles) were selected for symmetrical divergence by detecting 5 points (including one point in the center of the film and four symmetrical points around the film). The thickness of the films was measured with a spiral micrometer, and the average (accuracy 0.0001 mm) was recorded. The thickness of the film provides the necessary index basis for the subsequent film strength test. A computer-controlled electronic tensile testing machine (WDW-5, Shenzhen, China) was used to determine its tensile strength (TS) and elongation at break (E). Before testing, the sample film strip was placed in a sealed box with a relative stable humidity (50%) at room temperature (25 °C) for 48 h before testing. The longest distance between the upper and lower clamps was 50 mm, and the speed rate was 0.5 mm/s. All experiments were carried out in triplicate and the results were shown as average value ± SD. The tensile strength (TS) referred to the ratio of the maximum tensile tension (F) that the film could withstand before rupturing under the action of the axial tensile force and the product of the width (L) and thickness (D) of the film. The equation for calculating tensile strength was used as follows (1):

(1) $$\mathrm{TS}=\frac{F}{L\times D}$$

Here, TS corresponds to the tensile strength (MPa). F indicates the maximum tension (N) that the sample bears when it breaks. L and D are the width (mm) and the thickness of the sample (mm).

The calculation equation of elongation at break refers to (2):

(2) $$\mathrm{EB}=\frac{h-H}{H}\times 100\%$$

Here, EB corresponds to the elongation at break (100%). H and h are the length of the sample film before stretching (mm) and the stretched length of the sample film (mm). 

Finally, Statistical Product and Service Solutions (SPSS) was used to statistically analyze the significance of the experimental results. Different letters indicate significant differences in data.

### 2.6. Morphology Characterization by SEM

A field emission scanning electron microscope (SEM 230, FEI, Hillsboro, OR, USA) was used to observe the microstructure of the film surface. Films were immersed in liquid nitrogen, and they were brittle and easily fractured at extremely low temperatures, and the microstructures of their cross-section were observed. Before the scanning electron microscope test, the films were fixed on the silicon wafer by conductive silica gel. Meanwhile, the surface of the film was gold-plated to make it conductive to obtain clear scanned images.

### 2.7. Fourier Transforms Infrared (FTIR) Spectroscopy 

The films prepared above were dried and cut into pieces, and then mixed with potassium bromide, milled and pressed into pieces. The infrared spectrum absorption peak of the films was measured by Fourier infrared spectrometer (NEXUS 670, Waltham, MA, USA) [33,34,35].

### 2.8. Thermogravimetric Analysis (TGA)

The thermal properties of films were tested with a thermogravimetric analyzer (TGA WRT-12, Shanghai, China) in a nitrogen atmosphere with a flow rate of 25 mL/min. The test temperature increased from 25 °C to 800 °C at a heating rate of 10 °C/min, and the thermal curves were recorded [36].

### 2.9. Antibacterial Properties

*Escherichia coli* (ATCC 11775) and *Staphylococcus aureus* (ATCC 27735) are representative Gram-negative bacteria and Gram-positive bacteria. These two bacteria were used to evaluate the antibacterial properties of the films. Here, we judge whether the bacterial growth is in the logarithmic phase, which is determined by the bacterial growth curve. Use a 96-well culture plate to grow bacteria and count them. The growth curve is drawn with the culture time as the abscissa and the bacterial count as the ordinate. When the bacteria grow logarithmically, it is the logarithmic phase. Bacteria in the logarithmic phase (200 μL of 1 × 10^3^ CFU/mL) with the best vitality were chosen and cultured in the solid agar medium at 37 °C for 24 h. After the injection of a bacterial solution into the medium, the bacterial solution was homogeneously spread and then dried for use later. The films were placed on a clean bench for UV sterilization for 30 min and then were cut into small discs (Φ 15 mm) with a puncher. The discs were carefully pasted on the surface of the plate culture medium. Three pieces of each medium were attached to reduce the salt error. The plate was placed upside down in an incubator (37 °C, 24 h). Then the diameters of the inhibition zone were measured and photographed.

## 3. Results

### 3.1. Characterization of the Film 

#### 3.1.1. Process Optimization of Composite Films by Mechanical Properties

**Figure 2:**
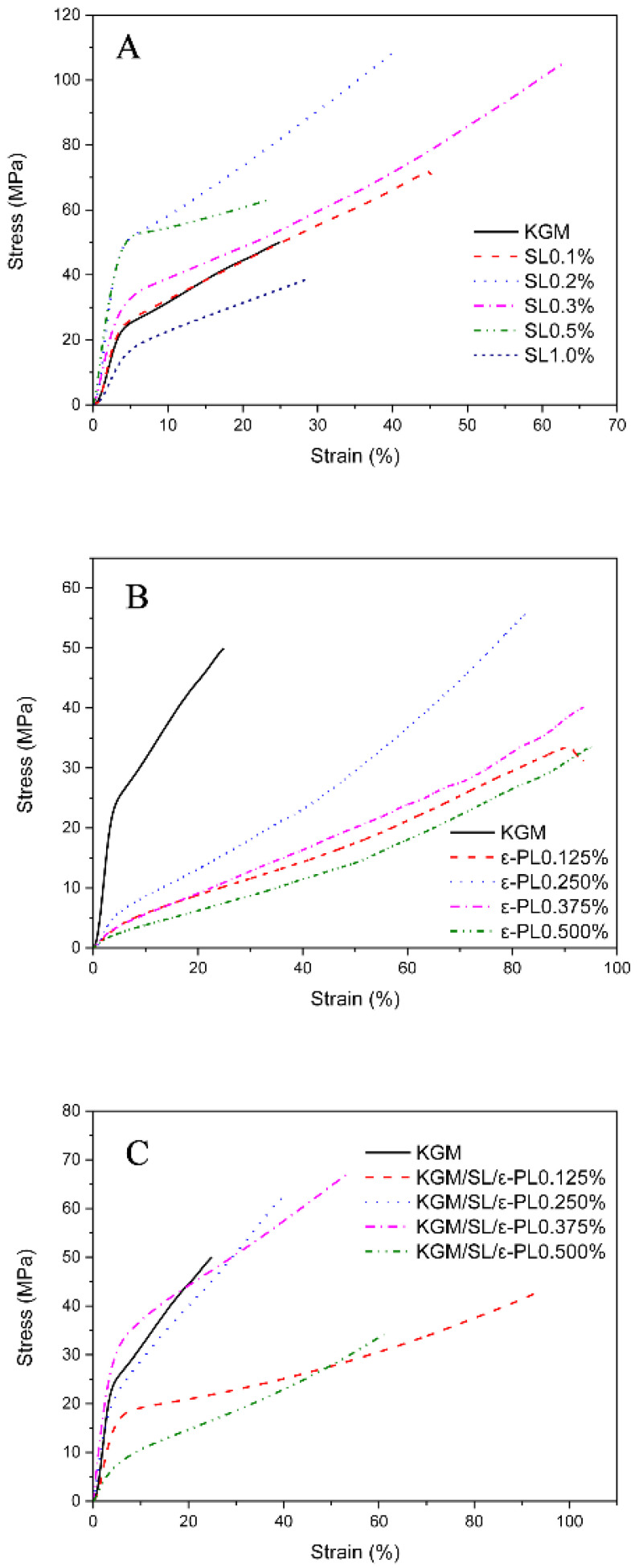
The stress–strain curves of the KGM/SL (**A**), KGM/ε-PL (**B**), KGM/SL/ε-PL (**C**) films with different components.

The crosslinking of the polymers of the film is shown in Figure 1, which explains the mechanical properties of the film to a certain extent. According to the research experience of our group, 1% KGM optimized gel concentration was used in composite films preparation. On basis of 1% KGM, films with varied SL concentrations of 0, 0.1, 0.2, 0.3, 0.5, 1.0% were prepared. The thickness of the film affects the tensile strength (Equation (1)), so the thickness of the film was measured as showed in Table 1. The tensile strength and elongation at break (shown in Table 1) of the films were obtained by the tensile strength test. SPSS statistical analysis results show that different letter superscripts indicate that the test results have significant differences and have reference significance. The stress–strain curves (shown in Figure 2A–C) have summarized the tensile strength variance of films with different combinations of KGM, SL and ε-PL. As showed in Figure 2A, films were prepared with stable KGM (1%, *w*/*v*) and varied SL amounts (0, 0.1, 0.2, 0.3, 0.5, 1.0%, *w*/*v*). From which, it could be observed that as the concentration of SL increased, the initial tensile strength increased and then decreased. We obtained the optimal concentration of SL. The maximum tensile strength is 108.55 ± 8.50 MPa (SL 0.2%), and the minimum is 38.56 ± 6.18 MPa (SL 1.0%) which might be that SL itself has a certain strength due to its rigid network. However, SL lacks flexibility, so that the concentration is too high and the brittleness becomes remarkable, which leads to a decrease in the elongation at the break of the film. In addition, the phenolic hydroxyl and sulfonic acid groups in SL enhance the hydrogen bonding force within the KGM molecular chains, thereby improving the mechanical strength of the original KGM film. At the same time, the electrostatic interaction between cationic polymer ε-PL and anionic polymer SL also further enhances the force between the components of the composite film. The results also confirmed that KGM/SL films with an SL concentration of 0.2% had better mechanical properties than other films with different SL concentrations.

It can be informed from Figure 2B that KGM/ε-PL films have no obvious advantage in tensile strength, but the elongation at the break of the film is greatly improved. As shown in Figure 2C, there are stress curves of films with increasing ε-PL concentration (0.125, 0.25, 0.375, 0.5%) but stable concentration of KGM (1%) and SL (0.2%). Too high a concentration of ε-PL will greatly facilitate interaction with KGM and SL to form an aggregate of small particles, which will affect the uniformity of the film; thus, the test is not performed. The mechanical strength of the KGM/SL/ε-PL composite film is much stronger than that of the pure KGM film (as control) with the minimum stress and strain. According to the control experiment, it reveals that SL greatly enhances the tensile strength of the film, while ε-PL greatly enhances the elongation at the break of the film. The results show that the optimized ratio of the matrix is 1% KGM, 0.2% SL, 0.375% ε-PL.

#### 3.1.2. Morphology Characterization

**Figure 3:**
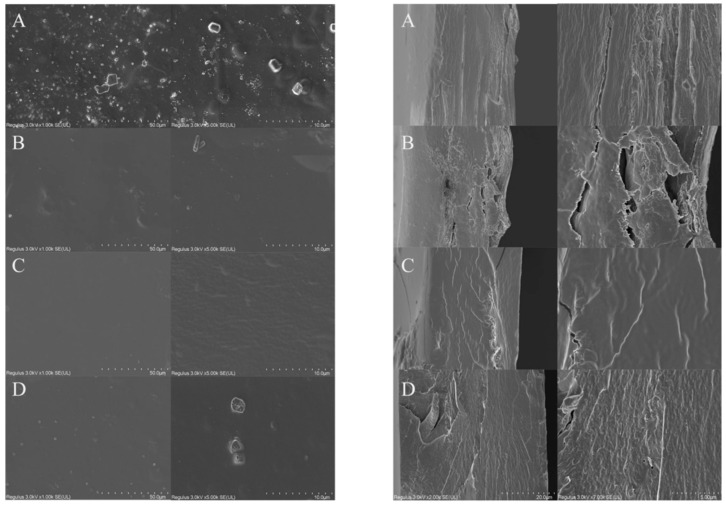
SEM images of the film surface (**left**) and cross section (**right**): (**A**) KGM (KGM 1%) film, (**B**) KGM/SL (KGM 1%, SL 0.2%) film, (**C**) KGM/ε-PL (KGM 1%, ε-PL 0.375%) film, (**D**) KGM/SL/ε-PL (KGM 1%, SL 0.2%, ε-PL 0.375%) film.

The microstructure of the films was observed by SEM (Figure 3). The surface (left) and cross-section (right) of the films were viewed with a magnification of 1.00 K and 5.00 K as showed in Figure 3 A, B, C, and D corresponding to KGM, KGM/SL, KGM/ε-PL and KGM/SL/ε-PL films, respectively. It could be observed from Figure 3A that the bubbles like particles of KGM were significantly more than those of KGM/SL, KGM/ε-PL and KGM/SL/ε-PL. Additionally, under the same stirring conditions, the KGM film appeared undissolved particles, which also confirmed that SL can enhance the dispersibility of the system. Under the same processing conditions, KGM had a strong affinity with water molecules due to the presence of hydrophilic acetyl groups and a large number of hydroxyl groups. The large molecular weight and a long chain of KGM making it difficult to remove bubbles. The addition of SL and ε-PL enable the solution to possess good fluidity and could be easier to remove bubbles. By observing the cross-section of the film, we found that the gap in the Figure 3B image is more obvious, which is related to the brittleness of SL found in our tensile test. The cross-section of the composite membrane seems denser, which is the same as the result of the tensile test.

#### 3.1.3. Fourier Transforms Infrared (FTIR) Spectroscopy 

**Figure 4:**
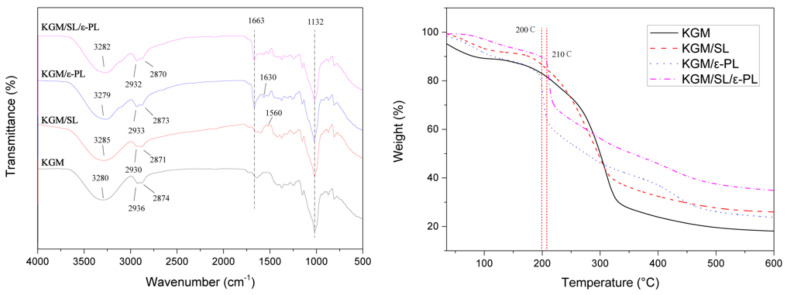
FITR and TGA curves of KGM (KGM 1%), KGM/SL (KGM 1%, SL 0.2%), KGM/ε-PL (KGM 1%, ε-PL 0.375%) and KGM/SL/ε-PL (KGM 1%, SL 0.2%, ε-PL 0.375%) films.

The chemical structure of KGM and KGM composite films was analyzed by FTIR (Figure 4). The spectrum of neat KGM shows some absorption peaks potentially sensitive to intermolecular interactions. These are bands at 3280 cm^−1^, assigned to the stretching of -OH groups. SL in KGM matrix leads to intensity variation of mentioned bands, which could be ascribed to the interaction between the -OH group on the SL surface and the hydroxyl groups of the KGM matrix. Slight modifications in the intensity of some peaks relating to SL are also visible at 1560 cm^−1^ (C-C stretching of the aromatic skeleton). In the case of KGM/ε-PL blending film, the two main characteristic peaks related to C-H were shifted towards higher wavenumbers (at 1630 cm^−1^) due to the interaction between -OH and -NH_2_ with bending vibration in KGM/ε-PL [37]. The absorption peak of C=O of the ε-PL structure was located at the wavelength of 1663 cm^−1^ conforming to the reference [38]. The shift of peak at around 2936 cm^−1^ to a lower frequency range may be due to hydrogen bonds among -OH, -SO_3_H and -NH_2_ in the films [39]. According to the analysis and comparison of the database, 2936 cm^−1^ and 2874 cm^−1^ in the KGM infrared spectrum represented the stretching vibration absorption peaks of -CH_2_- [40]. The absorption peak at 1132 cm^−1^ without obvious change could be assigned as the absorption peak of in-plane bending vibration of the alcoholic hydroxyl group.

#### 3.1.4. Thermogravimetric Analysis (TGA) 

The thermal behavior of KGM and KGM composite films was assessed by TGA (Figure 4) at a temperature ranging from 30 to 600 °C. The typical degradation of KGM is divided into three weight loss steps. The first step (in the 50–200 °C region) is related to the volatilization and evaporation of water. Water loss of KGM in the first step is due to its hydrophily [31]. In the second step, all films showed a large amount of mass loss at 200–325 °C deriving from thermal degradation of KGM, SL and ε-PL. The initial pyrolysis temperature of KGM, KGM/SL, and KGM/ε-PL was less than 200 °C, while the initial pyrolysis temperature of KGM/SL/ε-PL was approximately at 210 °C. The weight loss of the KGM film was greatly slowed down at around 330 °C [32]. The increase in the pyrolysis temperature of the KGM/SL/ε-PL composite film proves that the thermal stability of the film was improved, which may be due to the increase in the hydrogen bond force between -OH of SL, -NH_2_ of ε-PL and -OH of KGM [35]. The interactions including van der Waals forces between components of composite film were enhanced and finally exhibited by the enhancement of the thermal stability, mechanical strength [41].

#### 3.1.5. Antibacterial Properties

**Figure 5:**
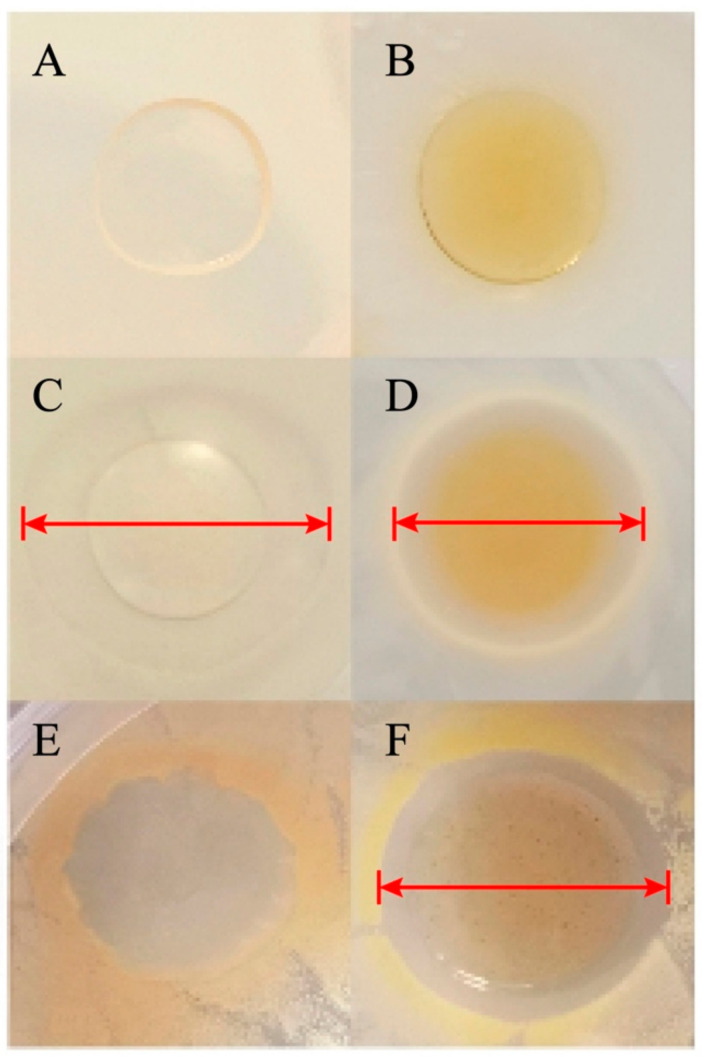
Antibacterial activity of films against *Escherichia coli* and *Staphylococcus aureus* in co-culturation mode: (**A**) KGM (KGM 1%) film (*E. coli*), (**B**) KGM/SL (KGM 1%, SL 0.2%) film (*E. coli*), (**C**) KGM/ε-PL (KGM 1%, ε-PL 0.375%) film (*E. coli*), (**D**) KGM/SL/ε-PL (KGM 1%, SL 0.2%, ε-PL 0.375%) film (*E. coli*), (**E**) KGM (KGM 1%) film (*Staphylococcus aureus*), (**F**) KGM/SL/ε-PL (KGM 1%, SL 0.2%, ε-PL 0.375%) film (*Staphylococcus aureus*).

The antibacterial activities of the film against *Escherichia coli* and *Staphylococcus aureus* were evaluated by 24 h co-culturation (Figure 5). Figure 5A–D refer to the inhibition of *E. coli* by KGM, KGM/SL, KGM/ε-PL and KGM/SL/ε-PL films respectively. It can be found that both KGM/ε-PL and KGM/SL/ε-PL films show obvious inhibition zones with inhibitory diameters of 26.87 ± 0.24 mm and 22.50 ± 0.31 mm (Figure 5C,D), while the KGM, KGM/SL films have no inhibitory diameter (Figure 5A,B) which meant that the added ε-PL has obvious antibacterial properties [42]. The final antibacterial activity of the composite film is weaker than that of KGM/ε-PL film due to the decreased mass proportion of ε-PL. As shown in the inhibition effects of KGM and KGM/SL/ε-PL films against *Staphylococcus aureus* are shown in Figure 5E,F, which revealed that the KGM film showed no antibacterial effect on *Staphylococcus aureus*, while an obvious inhibition zone is formed around the KGM/SL/ε-PL film with a diameter of 19.69 ± 0.36 mm. In summary, it is found that the KGM/SL/ε-PL composite film shows an advanced inhibitory effect on bacterial compared with other films. That is to say, the addition of ε-PL significantly improves the antibacterial effect of the composite film.

## 4. Conclusions

In this study, the tensile properties, thermal stability, and antibacterial properties of the KGM/SL/ε-PL composite film had been significantly improved by introducing SL and ε-PL. The tensile strength of the composite film is significantly increased to 105.97 ± 4.58 MPa (compared to 49.91 ± 5.88 MPa of the KGM film, *p* < 0.05). Meanwhile, different concentration gradients were used to screen the optimal component of KGM/SL and KGM/ε-PL films. By comparing with KGM, it is found that an appropriate SL concentration can increase the tensile strength and elongation at the break of the film. Moreover, the addition of ε-PL significantly increased the elongation at the break of the film. The screening results show that the optimized ratio of KGM/SL/ε-PL film is: KGM 1%, SL 0.2% and ε-PL 0.375%. Finally, the thermal stability of composite film is improved by introducing SL and ε-PL and has the strongest thermal stability under high-temperature pyrolysis conditions. The addition of ε-PL enabled the film to possess the expected antibacterial properties. It has proved that the composite film can be a good candidate for food packaging. Compared with the one-component pure KGM packaging film, the composite film has significantly enhanced mechanical properties, which helps to enhance the protection and applicability of food packaging. Enhanced thermal stability and antibacterial properties also provide the possibility to improve the durability of composite films in food packaging. At the same time, the use of natural biological materials makes the film more biodegradable, which is conducive to environmental protection and sustainable development and shows potential in the field of food green packaging. In addition, the preparation of composite films can be further improved. For example, enhance the fluidity of KGM to reduce bubbles, thereby improving the uniformity of film formation; further research to improve the air permeability, water solubility and oxidation resistance of the composite film; in-depth study of the mechanism of film polymer molecules to produce stronger mechanical performance biodegradable food packaging film.

## Figures and Tables

**Table 1:** Thickness and mechanical performance of films with different components.

Sample	Thickness (μm)	Tensile Strength (MPa)	Elongation at Break (%)
KGM	26.80 ± 1.30 ^i^	49.91 ± 5.88 ^d^	24.89 ± 4.51 ^e^
SL0.1%	33.20 ± 2.00 ^gh^	72.29 ± 5.10 ^c^	45.23 ± 4.52 ^d^
SL0.2%	38.20 ± 2.80 ^ef^	108.55 ± 8.50 ^a^	40.08 ± 0.56 ^d^
SL0.3%	41.40 ± 5.90 ^de^	105.19 ± 4.52 ^a^	62.81 ± 3.50 ^c^
SL0.5%	58.20 ± 1.60 ^b^	63.36 ± 3.50 ^c^	23.80 ± 3.04 ^e^
SL1.0%	70.00 ± 3.10 ^a^	38.56 ± 6.18 ^de^	28.45 ± 5.07 ^e^
PL0.125%	28.80 ± 3.00 ^hi^	33.40 ± 9.56 ^e^	93.54 ± 3.02 ^a^
PL0.250%	36.80 ± 3.30 ^efg^	40.13 ± 4.03 ^de^	71.87 ± 4.05 ^b^
PL0.375%	35.80 ± 3.60 ^fg^	42.38 ± 5.50 ^de^	93.57 ± 3.02 ^a^
PL0.500%	37.00 ± 3.40 ^efg^	33.55 ± 5.50 ^e^	95.01 ± 5.00 ^a^
KGM1	43.80 ± 4.40 ^cd^	70.21 ± 7.54 ^c^	70.03 ± 6.56b ^c^
KGM2	43.80 ± 4.40 ^cd^	89.41 ± 7.14 ^b^	91.67 ± 6.05 ^a^
KGM3	45.40 ± 5.10 ^cd^	105.97 ± 4.58 ^a^	95.71 ± 5.02 ^a^
KGM4	46.60 ± 2.40 ^c^	97.12 ± 7.00 ^ab^	91.47 ± 1.04 ^a^

Different superscript letters indicate a significant difference between the means (*p* < 0.05).
